# Comparison between APACHE II and POSSUM 2 scores in neurosurgical patients admitted to an ICU in Fortaleza, Brazil

**DOI:** 10.1186/cc12649

**Published:** 2013-06-19

**Authors:** AA Peixoto, MNR Teixeira Henderson, CAR Feijó, VN Araújo, ZC Teixeira, RE Magalhães, FA de Meneses

**Affiliations:** 1 Centro Universitário Christus - Unichristus, Papicu, Fortaleza, CE, Brazil; 2 Hospital Universitário Walter Cantídio - UFC, Papicu, Fortaleza, CE, Brazil; 3 Hospital Geral De Fortaleza - SESA, Papicu, Fortaleza, CE, Brazil

## Introduction

The need to stratify surgical risk is essential to assess the quality of care of patients undergoing intervention. The objective of this study was to compare APACHE II and POSSUM 2 severity scores in patients undergoing neurosurgery, in the immediate postoperative period.

## Methods

An observational prospective study with 155 patients admitted consecutively to the ICU of a tertiary hospital in Fortaleza, Brazil, from December 2011 to June 2012, after neurosurgical intervention. **Results **The population analyzed showed an average age of 48.0 ± 15.8 years, predominantly female (55.3%). Elective surgeries were more prevalent (92.1%), especially aneurysm clipping (14.6%) and resection of neoplasm (64.2%). At admission, 45 patients (27.6%) had at least one organ dysfunction. The APACHE II score mean was 9.7 ± 5.1, corresponding to the mean of predicted mortality of 14.0 ± 10.7%. The POSSUM 2 score showed a trend to be higher in patients that died, the mean of the physiological score being 17.7 (20.0 IQ: 13.8 to 24.3 vs. 16.5 IQ: 14 to 20, *P *= 0.416) and the mean of surgical score being 11.2 (13.0 IQ: 10.5 to 14.3 vs. 10.0 IQ: 9 to 12, *P *= 0.06), corresponding to a mean of predicted mortality of 7.3% (6.9 IQ: 3.9 to 14.6 vs. 4.0 IQ: 2.6 to 6.4, *P *= 0.08) and average morbidity of 29.6% (37.0% IQ: 21.9 to 61.8 vs. 21.1% IQ: 14.6 to 34.3, *P *= 0.06). The mortality rate was 6.57%, generating a standardized mortality rate of 0.47 for the APACHE II score and 0.90 for the POSSUM 2 score.

## Conclusion

Our study suggests that the POSSUM 2 score could be a useful tool in predicting mortality in neurosurgical patients admitted to an ICU. It was more accurate to identify the real mortality than the APACHE II score.

